# The Association of Dietary Macronutrient Quality and Mortality Among Patients With Liver Cirrhosis: A Prospective Cohort Study

**DOI:** 10.1002/hsr2.72936

**Published:** 2026-07-29

**Authors:** Fatemeh Javaheri‐Tafti, Zeinab Ghaeminejad, Leyli Zahra Bahreini Boroujeni, Niayesh Naghshi, Azita Hekmatdoost, Fereshteh Pashayee‐khamene, Sara Karimi, Saleheh Ahmadzadeh, Mehdi Saberifiroozi, Behzad Hatami, Zahra Yari

**Affiliations:** ^1^ Student Research Committee Shahid Beheshti University of Medical Sciences Tehran Iran; ^2^ Department of Clinical Nutrition and Dietetics, Faculty of Nutrition Sciences and Food Technology, National Nutrition and Food Technology Research Institute Shahid Beheshti University of Medical Sciences Tehran Iran; ^3^ Student Research Committee Ahvaz Jundishapur University of Medical Sciences Ahvaz Iran; ^4^ Liver and Pancreatobiliary Research Center, Digestive Disease Research Institute, Shariati Hospital Tehran University of Medical Sciences Tehran Iran; ^5^ Gastroenterology and Liver Diseases Research Center, Research Institute for Gastroenterology and Liver Diseases Shahid Beheshti University of Medical Sciences Tehran Iran; ^6^ Department of Nutrition Research, National Nutrition and Food Technology Research Institute and Faculty of Nutrition Sciences and Food Technology Shahid Beheshti University of Medical Sciences Tehran Iran

**Keywords:** cirrhosis, diet quality index, macronutrients, mortality, MQI

## Abstract

**Background and Aims:**

Cirrhosis, an end‐stage manifestation of chronic liver disease, is a leading contributor to morbidity and mortality worldwide. Diet quality is an important component in determining the prognosis of various chronic diseases. In this prospective cohort study, we aimed to investigate the association between the macronutrient quality index (MQI) and mortality risk among patients with liver cirrhosis.

**Methods:**

In this prospective cohort study, 121 recently diagnosed cirrhotic patients were monitored for 60 months. The dietary intake of patients was evaluated using a 168‐item food frequency questionnaire, after which MQI and its sub‐indices including carbohydrate quality index (CQI), fat quality index (FQI), and protein quality index (PQI) were calculated. Crude and multivariable‐adjusted hazard ratio (HR) with 95% confidence interval (CI) were estimated based on cox proportional hazard models.

**Results:**

The findings showed that a higher MQI score was significantly associated with a 65% reduction in risk of mortality after adjusting for potential confounders (p trend = 0.024). Also, CQI (HR = 0.32, 95% CI = 0.1–0.97, p trend=0.026) and FQI (HR = 0.25, 95% CI = 0.07–0.9, p trend=0.023) indicated significant reverse associations, while PQI (HR = 0.33, 95% CI = 0.1–1.1, p trend=0.122) failed to show a significant association with mortality risk.

**Conclusion:**

A thorough evaluation of MQI and its sub‐indices and mortality risk in patients with cirrhosis revealed that improving the quality of macronutrient intake could considerably increase survival in cirrhotic patients.

## 1 Introduction

Cirrhosis is the terminal stage of chronic liver disease progression [1]. Liver diseases have demonstrated a concerning upward trend in prevalence worldwide over recent decades. This surge has positioned them as a leading contributor to both morbidity and mortality. The global burden of disease (GBD) project clarified this issue's dimension. In Iran, for instance, GBD data revealed that the year 2017 saw nearly 5,400 fatalities caused by cirrhosis and other chronic liver diseases [2]. This amounts to 8.12 deaths per 100,000 people, which is the age‐standardized mortality rate. Also, cirrhosis complications are estimated to be responsible for half of the global deaths attributed to liver disease, translating to roughly 1 million fatalities annually [3]. Metabolic dysfunction–associated steatotic liver disease (MASLD), previously known as non‐alcoholic fatty liver disease (NAFLD), often linked to obesity and metabolic syndrome (MetS), has become a significant contributor to the growth of cirrhosis cases globally [4, 5].

Lifestyle interventions, specially dietary modifications, have emerged as first‐line therapy for MASLD [6]. Healthy dietary practices include the regular consumption of fruits, vegetables, legumes, whole grains, and seafood, while moderating the intake of dairy, red meat, processed meat, and sweets [7]. Diet quality is crucial in assessing the risk of chronic diseases and is often assessed using varius indices [8, 9]. One such index is the macronutrient quality index (MQI), which assesses the quality of macronutrients, including proteins (PQI), carbohydrates (CQI), and fats (FQI) [10]. The macronutrient quality index was developed in 2022 to assess the overall quality of macronutrients in a diet by giving equal weight to each of the three macronutrients [11]. These indices play a significant role in both research and clinical applications, aiding in the evaluation of dietary intakes and their impact on health outcomes [10].

In a cross‐sectional study in Iran, the MQI served as a predictor for MetS and its components [12]. In the SUN cohort, data have revealed that adherence to a diet with a high MQI score is associated with a lower risk of overweight and obesity [13]. A case‐control research indicated that greater adherence to a diet with higher scores on the diet quality index‐international (DQI‐I) and the diet quality index‐revised (DQI‐R) was correlated with reduced likelihood of NAFLD. An elevated score of the DQI‐I and DQI‐R indicated a diet rich in advantageous food categories and limited in harmful dietary elements, perhaps aiding in the prevention of metabolic diseases such as MASLD [8]. A study showed that individuals who followed a diverse dietary pattern had lower odds of being diagnosed with MASLD, suggesting that dietary diversity may prevent this condition [14]. Another study concluded that a better quality diet, reflected by higher scores of DQI‐I and higher intakes of vegetables, legumes, and fruits, was related to reduced odds of MASLD among the Chinese population [15]. These results demonstrated that an elevated score of any component of the DQI correlates with a decreased risk of mortality from chronic liver disease (CLD). Also, following a diet with higher quality may help reduce the incidence of liver cancer and mortality rates from CLD [16]. Although, the results of recent study showed that there was no relationship between the CQI and MetS or its components [17]. Therefore, the results in this area are contradictory and not yet conclusive.

To our knowledge, there are no prospective studies that have investigated the association between the MQI and mortality in patients with liver cirrhosis. Therefore, we intended to prospectively assess the relationship between MQI and its dub indices with mortality rates among cirrhotic patients, during a 5‐year follow‐up phase.

## 2 Methods and Materials

### 2.1 Study Population

The methodology of this cohort study has been fully described elsewhere [18] except that the follow‐up period in the present study was 60 months (until April 30, 2024). Further details are briefly provided here. This cohort study was designed to monitor newly diagnosed outpatients with cirrhosis. 166 patients were initially enrolled in the study, of which 45 were excluded from the final analysis for the following reasons: cancer diagnosis during the first year, lacked comprehensive general lifestyle or dietary information, exhibited excessive or insufficient energy intake, had an extreme body mass index. Upon joining the cohort, the participants were monitored yearly. Participants got yearly telephone calls to complete follow‐up questionnaires about the occurrence of death or any medical incident. Mortality and survival rates were assessed at the end of 5 years. Ultimately, 121 subjects (38 females and 83 males) were considered eligibale for the analysis. The research protocol received approval from the ethical committee of the National Nutrition and Food Technology Research Institute (IR.SBMU.NNFTRI.1396.186.). All participants were apprised of the research, and formal permission forms were obtained.

### 2.2 Exposure Assessment

Dietary data were collected at baseline using a valid 168‐item food frequency questionnaire (FFQ) administered via in‐person interviews [19]. The MQI was derived from dietary intake data obtained through the FFQ. It was calculated based on the sum of three sub‐indices including CQI, FQI, and the Healthy Plate Protein Source Quality Index (HPPQI). CQI is a composite score reflecting carbohydrate quality, calculated as the sum of four equally weighted sub‐scores derived from glycemic index (GI), total dietary fiber intake (g/day), the ratio of whole grains to total cereals (whole grains + refined cereals + products prepared with refined flours), and the ratio of solid carbohydrates to total carbohydrates (liquids + solids). The FQI was computed using the formula: (monounsaturated + polyunsaturated)/(saturated + trans fatty acids). HPPQI describes the ratio of consumption of healthy sources of protein to unhealthy sources based on the following ratio: (seafood + poultry + pulses + nuts)/(red and processed meats + cheese). The MQI varies from 3 to 15, with larger values indicating greater macronutrient quality [11].

### 2.3 Potential Confounders

At enrollment, data on basic characteristics, nutritional status, anthropometric measurements, and liver disease severity were collected. The prognosis and severity of liver cirrhosis in participants were evaluated using the Child‐Pugh score and the model for End‐Stage Liver Disease (MELD) score [20]. The child‐Pugh score was calculated based on five parameters: serum albumin, serum bilirubin, prothrombin time, presence of ascites, and encephalopathy. Each parameter was scored on a scale of 1 to 3 (higher scores indicating more severe disease). This score classified patients into 3 classes (A, B, and C). The MELD score was determined using the following formula: (3.78 × Ln (total bilirubin, mg/dl))+(11.2 × Ln (INR)) + (9.57 × Ln (creatinine, mg/dl))+6.43 [20]. Also, subjective global assessment (SGA) was obtained using the Destky et al. study [21]. In accordance with this evaluation, participants were split up into three groups: A: indicates adequate nutrition, B: moderate malnourishment, and C: severe malnourishment.

### 2.4 Statistical Analysis

All statistical analyses were conducted using SPSS software (version 19; SPSS Inc., Chicago, IL, USA). Participants were categorized into quartiles based on the macronutrient quality index. Baseline characteristics and dietary intakes were presented as means ± standard deviation (SD) for continuous variables and percentages for categorical variables, which were analysed using a one‐way analysis of variance (ANOVA) and Chi‐squared (*χ* ^2^) test, respectively. Cox proportional hazards regression models were applied to estimate multivariable‐adjusted hazard ratios (HR) and 95% confidence intervals (CI) for risk of mortality associated with the quartiles of MQI, as well as its sub indices. Potential confounders were adjusted in three sequential models: Model 1 was adjusted for age and sex; Model 2 additionally adjusted for energy intake, BMI, smoking and alcohol use (yes, no); and Model 3 further adjusted for cirrhosis etiology (virus, autoimmune, other), subjective global assessment (A, B, C), MELD score (continuous), and Child‐Pugh classification (A, B and C). All statistical tests were two‐sided, and a *p*‐value < 0.05 was considered statistically significant. This study was reported in accordance with the STROBE guidelines for observational studies.

## 3 Results

The mean age ± SD of participants at baseline was 54.8 ± 11.9 years, and in total, 68.6% were men. During 60 months of follow‐up, we documented 50 deaths. According to the table, there were no significant differences in age, etiology of cirrhosis, alcohol and smoking habits, anthropometric parameters (weight, height and body mass index), and subjective global assessment among the participants in MQI quartiles. However, cirrhosis seveity and prognosis (MELD score and Child‐Pugh) were significantly improved from first to last quartile of MQI. The baseline characteristics of the participants according to the macronutrient quality index quartiles are shown in Table 1.

**Table 1:** Characteristics of participants based on quartiles of MQI.

	Quartile of total MQI				
	Q1	Q2	Q3	Q4	*p* value
Men, %	75.9	70.8	75.9	42.1	0.054
Age (y)	56.1 ± 11.02	56.8 ± 10.4	55.6 ± 12.9	50.9 ± 10.27	0.345
Etiology of cirrhosis					0.320
Virus	56	62.5	50	36.8	
Autoimmune	32	20.8	46.2	42.1	
Other	12	16.7	3.8	21.1	
MELD score	14.8 ± 5.1	13.3 ± 5.6	12.4 ± 5.2	9.9 ± 3.4	0.030
Child‐Pugh category					0.034
A	47.6	57.1	76.2	88.2	
B, C	52.4	42.9	23.8	11.8	
Alcohol drinker	28.6	13	23.1	26.3	0.593
Smoker, %	32.1	39.1	50	21.1	0.218
Weight, kg	73.3 ± 14.3	72.9 ± 15.6	71.7 ± 125.7	76.5 ± 23.1	0.815
Height, cm	165.2 ± 8.8	165.4 ± 10	165.2 ± 6.1	162.8 ± 8.9	0.731
Body mass index, kg/m^2^	26.9 ± 4	26.8 ± 4.8	26.4 ± 5.4	28.6 ± 7.1	0.559
Subjective global assessment					0.307
A	34.5	16.7	27.6	36.8	
B	41.4	75	58.6	47.4	
C	24.1	8.3	13.8	15.8	

*Note:* Values are means ± SDs for continuous variables and percentages for categorical variables. ANOVA for quantitative variables and *χ* ^2^ test for qualitative variables.

According to Table 2, there were no significant differences in dietary intakes of participants across quartiles of MQI. Calorie intake showed a slight increase from the first to the fourth quartile, but it was not significant.

**Table 2:** Dietary intakes of study participants by quartiles of Macronutrient Quality Index (MQI).

	Quartile of total MQI				
	Q1	Q2	Q3	Q4	*p* value
Energy (kcal/d)	1939 ± 625	2014 ± 748	2069 ± 645	2323 ± 634	0.307
Protein (% TEI)	14.4 ± 2.3	13.5 ± 3.5	14.9 ± 2.9	14.8 ± 2.8	0.441
Carbohydrate (% TEI)	60.8 ± 7.4	59.1 ± 9.4	59.3 ± 6.3	58 ± 8.5	0.739
Fat (% TEI)	27.7 ± 6.6	29.2 ± 9.9	27.8 ± 5.8	28.4 ± 8.7	0.922
PUFAs (g/d)	13.6 ± 6.7	16.6 ± 6.4	14.3 ± 6.9	15.2 ± 5.8	0.441
MUFAs (g/d)	18.1 ± 7.7	22.9 ± 9	19.8 ± 7.2	22.3 ± 7.6	0.156
SFAs (g/d)	16.1 ± 5.9	16.4 ± 7.1	16.8 ± 6.2	17.6 ± 4	0.859
Fiber (g/1000 Kcal)	13.7 ± 5.2	12.5 ± 4.6	13.7 ± 3.8	14.7 ± 4	0.471
Vegetables	244 ± 164	237 ± 170	270 ± 167	249 ± 168	0.919
Fruits	309 ± 145	329 ± 182	337 ± 173	344 ± 172	0.939
Meats	63 ± 41	83 ± 54	74 ± 46	75 ± 44	0.509
Cereals	365 ± 128	398 ± 177	395 ± 167	399 ± 161	0.853
Dairy	230 ± 138	188 ± 153	223 ± 134	251 ± 130	0.590

*Note:* Values are means ± SDs for continuous variables and percentages for categorical variables. ANOVA for quantitative variables and *χ* ^2^ test for qualitative variables.

Hazard ratios (HR) and 95% CI for the association between quartiles of the MQI and its sub indices are shown in Table 3. Increasing the quality of macronutrients, in whole and in each component, were significantly associated with a reduction in the number of deaths. MQI, FQI, and CQI showed an inverse and significant relationship with the risk of mortality, but the association between mortality and PQI, although inverse, was not statistically significant. Using different models to adjust for confounders did not change the significance level.

**Table 3:** Hazard ratios (HR) and 95% confidence intervals (CI) for the association between quartiles of the Macronutrient Quality Index (MQI), Carbohydrate Quality Index (CQI), Fat Quality Index (FQI), Protein Quality Index (PQI), and all‐cause mortality.

MQI	Q1	Q2	Q3	Q4	*p* trend
No. of deaths	21	15	9	5	0.009
Model 1	ref	0.61 (0.29–1.3)	0.46 (0.21–1)	0.26 (0.09–0.77)	0.006
Model 2	ref	0.58 (0.21–1.55)	0.54 (0.22–1.3)	0.34 (0.1–1.1)	0.049
Model 3	ref	0.77 (0.34–0.91)	0.67 (0.24–0.86)	0.35 (0.07–0.7)	0.024
**CQI**					
No. of deaths	26	12	7	5	0.037
Model 1	ref	0.56 (0.27–1.2)	0.48 (0.2–1.2)	0.29 (0.09–0.98)	0.014
Model 2	ref	0.62(0.29–1.3)	0.51 (0.11–0.98)	0.3 (0.09–0.99)	0.018
Model 3	ref	0.7 (0.3–1.5)	0.52 (0.2–1.3)	0.32 (0.1–0.97)	0.026
**FQI**					
No. of deaths	24	12	9	5	0.017
Model 1	ref	0.57 (0.28–1.18)	0.45 (0.2–1.05)	0.21 (0.06–0.7)	0.003
Model 2	ref	0.59 (0.23–1.3)	0.57 (0.23–1.3)	0.26 (0.08–0.9)	0.016
Model 3	ref	0.69 (0.33–1.47)	0.65 (0.25–1.6)	0.25 (0.07–0.9)	0.023
**PQI**					
No. of deaths	21	14	10	5	0.042
Model 1	ref	0.85 (0.41–1.75)	0.78 (0.3–1.7)	0.34 (0.1–1.1)	0.089
Model 2	ref	0.78 (0.37–1.6)	0.93 (0.4–2.2)	0.37 (0.1–1.2)	0.157
Model 3	ref	0.9 (0.42–1.9)	0.91 (0.41–2.2)	0.33 (0.1–1.1)	0.122

*Note:* Cox proportional hazards regression models for estimating HRs and 95% CIs. Model 1: adjusted for age and sex. Model 2: additionally adjusted for energy intake, BMI, smoking and alcohol. Model 3: additionally adjusted for etiology, SGA, MELD, and child.

In the fully adjusted model (Model 3), the risk of mortality was 65% lower for participants in the highest quartile of MQI compared with the lowest quartile (HR = 0.35, 95% CI: 0.07–0.7, *p* trend = 0.024). Also, with increasing CQI scores, a significant inverse association with a decrease in mortality risk was observed, such that in the third model, the risk of mortality in the last quartile was 68% lower than the reference group (HR = 0.32, 95% CI: 0.1–0.97, *p* trend = 0.026). Higher FQI was also significantly associated with a lower risk of mortality. The HR for Q4 in the third model was 0.25 (HR = 0.25, 95% CI: 0.07–0.9, *p* trend = 0.023), representing a 75% lower risk of mortality. Increasing PQI score was also associated with a reduced risk of mortality, although the results were not statistically significant in the models (HR = 0.33, 95% CI: 0.1–1.1, *p* trend = 0.122).

**Figure 1:**
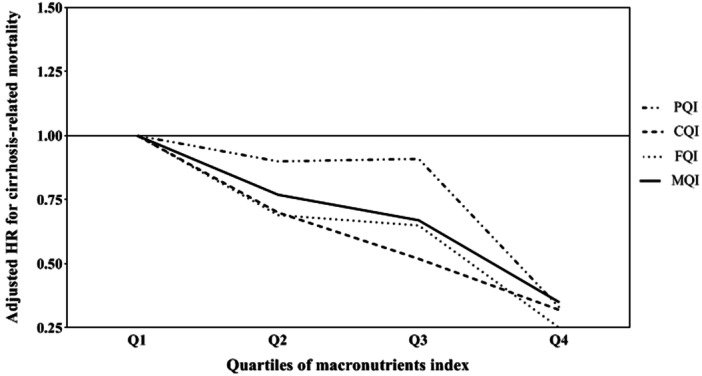
Adjusted hazard ratio for mortality risk across macronutrient index scores and its subsets.

The inverse relationship between the MQI and its subscales with mortality, after adjusting for all confounders, is depicted in Figure 1.

## 4 Discussion

This cohort research was carried out to examine the association between MQI and death rates in patients with liver cirrhosis throughout a 5‐year follow‐up period. Our research results indicated significant reverse associations between the MQI, FQI, and CQI and the mortality risk in patients with liver cirrhosis. This relationship was inverse but non‐significant for PQI. The results indicated that, after adjusting for possible confounders, mortality decreased by 65% for those in the highest quartile of MQI compared to those in the lowest quartile.

To our knowledge, limited research has addressed the association between MQI and LCD, and has mainly focused on DQI. Studies examining the association between DQI and NAFLD or MASLD, have shown that following a diet with a high DQI score and high diversity may reduce the risk of these diseases [8, 14]. Also, studies suggested that there is an association between various DQI scores including healthy eating index (HEI)‐2010, alternative HEI‐2010, alternate mediterranean diet (aMED) and dietary approaches to stop hypertension (DASH) with CLD, HCC, and MASLD, as well as cirrhosis. The findings of the multiethnic cohort study revealed significant negative associations between these four DQI scores and CLD, however an inverse relationship for HCC was not significant [16]. Another cohort study demonstrated that higher HEI‐2015 and DASH scores were negatively correlated with NAFLD, indicating that an improved DQI may reduce the risk of NAFLD, particularly benefiting those with both NAFLD and cirrhosis [22]. The inverse correlation between the risk of NAFLD, MASLD, and CLD in general with DQI has been attributed to several factors. One possible mechanism is the presence of fiber, protein, MUFA, omega‐3, calcium, iron, vitamin C, vitamin A, and polyphenols in a high‐quality diet, which have anti‐inflammatory and antioxidant properties [23, 24, 25, 26]. Diets with high DQI have also been shown to have a protective role against overweight, obesity, insulin resistance, and MetS, all of which are risk factors for CLD, due to their anti‐inflammatory and antioxidant properties [23, 27, 28].

Limited studies have also specifically addressed the MQI, as it has only recently been introduced. In the SUN cohort study, findings indicate that there is an inverse association between high‐quality macronutrient scores and all‐cause mortality. This association was determined for the MQI and all of its sub‐indices, however only the CQI showed a significant inverse relationship with mortality rate [29]. The results of the Mashhad PERSIAN cohort cross‐sectional study showed that greater adherence to the MQI was associated with a reduced risk of MetS and its related components, especially central obesity, increased blood glucose, and high triglyceride levels [12]. In a Mediterranean cohort, researchers found that the participants with the greatest MQI had a reduced incidence of overweight and obesity, and the association was significant [13]. Also, in a prospective study conducted in Tehran, individuals in the highest tertile of MQI and CQI exhibited a reduced incidence of type 2 diabetes compared to those in the lowest tertile [30]. As mentioned, a high‐quality diet is rich in antioxidant and anti‐inflammatory compounds that can reduce inflammation, improve endothelial function, and increase insulin resistance, all of which contribute to the development and progression of liver disease. Therefore, it can be supposed that diets with a high MQI score have anti‐inflammatory, antioxidant and anti‐atherogenic properties. These diets can also reduce the risk of developing, worsening and mortality associated with CLD by reducing visceral fat, improving blood sugar control and reducing hyperinsulinemia [31, 32, 33].

Also, a few limited studies have examined the MQI sub‐indices and their relationship with metabolic abnormalities, such as obesity, diabetes, and MetS, which significantly increase the risk of developing NAFLD and its progression to cirrhosis [34]. The present study showed a protective association of CQI with mortality in cirrhotic patients. Consistently, the results of the prospective Tehran Lipid and Glucose Study indicated that adherence to a diet characterized by a higher CQI and PQI score was significantly linked to a decreased risk of MetS in adults [35]. In contrast, a cross‐sectional research examined the relationship between meal‐specific CQI and MetS in Iranian adults. The results did not demonstrate a significant correlation between CQI and MetS or its components, even after adjusting for potential confounders [17]. High‐CQI diets, due to their high content of whole grains, high fiber, vitamins, and minerals, appear to reduce the risk of metabolic abnormalities [36]. High‐CQI diets, due to their low GI, are associated with significantly increased adiponectin levels and decreased levels of inflammatory biomarkers including IL‐6, CRP, and TNF‐α. These metabolic changes may reduce triglycerides, total cholesterol, blood glucose, and blood pressure [37, 38]. On the other hand, foods with a low glycemic index and low glycemic load are digested and absorbed more slowly, thus preventing a sudden surge in blood sugar and insulin secretion [39]. Therefore, following these diets is associated with reduced insulin resistance, oxidative stress, and inflammation, and may improve dyslipidemia [40, 41].

The present research found that a greater FQI was significantly correlated with reduced mortality rates. In line with these results, a cross‐sectional study revealed that each dietary lipid can exert distinct effects on MetS components in individuals with a high risk of CVD, and that dietary fat consumption is linked with an increased risk of hyperglycemia [42].

Another finding of our study was that higher PQI scores were associated with reduced mortality, although it was not statistically significant. This suggests the protective association of white meat and plant protein, as well as the detrimental association of red and processed meat. Consistently, a large population‐based study found a significant correlation between high animal protein intake and NAFLD in an overweight elderly Caucasian population. Both total and animal protein were associated with a higher risk of NAFLD after adjusting for sociodemographic, lifestyle, and metabolic confounders. Protein from vegetables had no correlation with NAFLD [43]. The presence of compounds such as nitrate, nitrite, and heme iron in red and processed meats may explain the association between PQI and CLD [44]. Heme may induce oxidative stress and insulin resistance [45]; nitrate and nitrite have also been reported to be associated with metabolic abnormalities [46]. Heme, nitrate, and nitrite in red meat have been linked to an increased risk of chronic diseases in a cohort study [47]. On the other hand, this association could be attributed to dietary acidity, as diets with a high acid load can reduce insulin sensitivity and pancreatic β‐cell responsiveness. This cascade of events may lead to type 2 diabetes, MASLD, and other liver diseases [48].

To the best of our knowledge, this cohort is the first to examine the relationship between MQI and mortality in patients with liver cirrhosis. The 5‐year follow‐up of this study is another strength. It is important to take into consideration the limitations of our research, First, the relatively limited small sample size of this study may reduce the statistical power and precision of the estimates. Therefore, the findings should be interpreted cautiously and confirmed in larger prospective cohorts. Second, dietary intake was assessed at baseline using a validated FFQ, which like all self‐reported, may encompass recall bias and measurement error. In addition, a single dietary assessment may not reflect potential changes in dietary patterns over time, leading to possible misclassification. Third, loss to follow‐up during the study may have introduced attrition bias. In addition, as in many epidemiological studies, residual confounding from unmeasured variables cannot be excluded. Fourth, due to the observational design, this study cannot established causality. Additionally, It is also possible that patients with greater disease severity altered their dietary behaviors, which may have contributed to reverse causation. Fifth, as the study population was derived from a specific regional cohort in Iran, the generalizability of the findings to other populations may be limited. Conducting clinical trials to assess variations in complications and mortality would strengthen our research.

## 5 Conclusion

In conclusion, a comprehensive assessment of the MQI, its sub‐indices, and mortality rate in patients with cirrhosis in our study revealed that improving the quality of dietary macronutrients may be associated with improved survival in cirrhotic patients. Further large‐scale prospective studies and randomized clinical trials are needed to confirm these findings and to determine whether improvements in macronutrient quality are associated with better survival outcomes in patients with liver cirrhosis.

## Author Contributions

**Fatemeh Javaheri‐Tafti:** project administration, writing – original draft. **Zeinab Ghaeminejad:** project administration, writing – original draft. **Leyli Zahra Bahreini Boroujeni:** project administration, writing – original draft. **Niayesh Naghshi:** methodology, writing – original draft. **Azita Hekmatdoost:** conceptualization, writing – review and editing. **Fereshteh Pashayee‐khamene:** methodology. **Sara Karimi:** methodology. **Saleheh Ahmadzadeh:** methodology. **Mehdi Saberifiroozi:** methodology. **Behzad Hatami:** methodology. **Zahra Yari:** conceptualization, formal analysis, writing – original draft, writing – review and editing.

## Funding

The authors have nothing to report.

## Ethics Statement

National nutrition and Food Technology Research Institute (NNFTRI) ethics committee approved the study protocol (Ir.sbmu.nnftri.1396.186.). All participants provided written informed consent and were informed about the study. All authors have read and approved the final version of the manuscript. Zahra Yari had full access to all the data in the study and takes responsibility for the integrity of the data and the accuracy of the data analysis. The corresponding author affirms that this manuscript is an honest, accurate, and transparent account of the study being reported; that no important aspects of the study have been omitted; and that any discrepancies from the study as planned have been explained.

## Conflicts of Interest

The authors declare no conflicts of interest.

## AI Disclosure

AI tools were used only for language editing and were not used for data analysis, interpretation, or decision‐making.

## Data Availability

The data supporting the findings of this study are available from the corresponding author on reasonable request.
